# Relationship between retinal vessel tortuosity and oxygenation in sickle cell retinopathy

**DOI:** 10.1186/s40942-019-0198-3

**Published:** 2019-11-18

**Authors:** Maziyar M. Khansari, Sarah L. Garvey, Shayan Farzad, Yonggang Shi, Mahnaz Shahidi

**Affiliations:** 1 Department of Ophthalmology, University of Southern California, 1450 San Pablo Street, Los Angeles, CA 90033-6103 USA; 2 Stevens Neuroimaging and Informatics Institute, Keck School of Medicine of USC, Los Angeles, CA USA; 3 College of Medicine, University of Illinois at Chicago, Chicago, IL USA

**Keywords:** Retina, Sickle cell retinopathy, Tortuosity, Oxygenation, Image analysis

## Abstract

**Background:**

Reduced retinal vascular oxygen (O_2_) content causes tissue hypoxia and may lead to development of vision-threatening pathologies. Since increased vessel tortuosity is an early sign for some hypoxia-implicated retinopathies, we investigated a relationship between retinal vascular O_2_ content and vessel tortuosity indices.

**Methods:**

Dual wavelength retinal oximetry using a commercially available scanning laser ophthalmoscope was performed in both eyes of 12 healthy (NC) and 12 sickle cell retinopathy (SCR) subjects. Images were analyzed to quantify retinal arterial and venous O_2_ content and determine vessel tortuosity index (VTI) and vessel inflection index (VII) in circumpapillary regions. Linear mixed model analysis was used to determine the effect of disease on vascular O_2_ content, VTI and VII, and relate vascular O_2_ content with VTI and VII. Models accounted for vessel type, fellow eyes, age and mean arterial pressure.

**Results:**

Retinal arterial and venous O_2_ content were lower in SCR (O_2A_ = 11 ± 4 mLO_2_/dL, O_2V_ = 7 ± 2 mLO_2_/dL) compared to NC (O_2A_ = 18 ± 3 mLO_2_/dL, O_2V_ = 13 ± 3 mLO_2_/dL) subjects (p < 0.001). As expected, O_2_ content was higher in arteries (15 ± 5 mLO_2_/dL) than veins (10 ± 4 mLO_2_/dL) (p < 0.001), but not different between eyes (OD: 12 ± 5 mLO_2_/dL; OS:13 ± 5 mLO_2_/dL) (p = 0.3). VTI was not significantly different between SCR (0.18 ± 0.07) and NC (0.15 ± 0.04) subjects, or between arteries (0.18 ± 0.07) and veins (0.16 ± 0.04), or between eyes (OD: 0.18 ± 0.07, OS:0.17 ± 0.05) (p ≥ 0.06). VII was significantly higher in SCR (10 ± 2) compared to NC subjects (8 ± 1) (p = 0.003). VII was also higher in veins (9 ± 2) compared to arteries (8 ± 5) (p = 0.04), but not different between eyes (OD: 9 ± 2; OS: 9 ± 2) (p = 0.2). There was an inverse linear relationship between vascular O_2_ (13 ± 5 mLO_2_/dL) content and VII (9 ± 2) (β = −0.5; p = 0.02).

**Conclusions:**

The findings augment knowledge of relationship between retinal vascular oxygenation and morphological changes and potentially contribute to identifying biomarkers for assessment of retinal hypoxia due to SCR and other retinopathies.

## Introduction

The retina has the highest rate of oxygen consumption per unit weight compared to other human tissues [1], thus requiring continuous delivery of oxygen and nutrients to maintain normal function. A major vision-threatening complication of sickle cell disease (SCD) is sickle cell retinopathy (SCR) [2, 3], which is associated with retinal capillary occlusion, ischemia, and neovascularization [5]. It was recently shown that retinal blood flow increases in response to reduced vessel oxygenation in SCR [4]. Also, increased tortuosity of small retinal vessels imaged by optical coherence tomography angiography (OCTA) in SCR has been demonstrated [6–8]. Variations in retinal vessel tortuosity may be due to changes in the tone of smooth muscles located on the vessel walls which are influenced by blood gas, mediators and metabolism [9]. Although alterations in retinal vessel tortuosity have been reported in hypoxia-implicated retinopathies [10–13], a relationship between vessel tortuosity and tissue oxygenation has not been reported. Such knowledge may help advance the understanding of mechanisms that lead to morphological changes in the retinal vasculature and potentially contribute to identifying vascular biomarkers for SCR characterization.

The purpose of the current study was to test the hypothesis that decreased retinal vascular oxygenation is associated with increased vessel tortuosity in healthy and SCR subjects. Oxygen content of major retinal vessels was quantified by our previously validated oximetry technique [14]. Since there is no universal agreement on which tortuosity measure is the best [15], in the current study vessel tortuosity was assessed by 2 metrics, namely vessel tortuosity index (VIT) and vessel inflection index (VII) as measured using our previously published method [6].

## Materials and methods

### Subjects

The study was approved by an institutional review board of the University of Illinois at Chicago. The study was explained to the subjects and written informed consents were obtained in accordance to the Tenets of Declaration of Helsinki. The cohort consisted of 12 (5 male and 7 female) heathy control (NC) and 12 (3 male and 9 female) SCR (11 stage 2 and 1 stage 3) subjects. All subjects had participated in our previous study which involved blood flow imaging and oximetry using different instruments [4]. Data from both eyes of subjects were included in the study. Eight, 3, and 1 of SCR subjects had hemoglobin SS, SC, and hemoglobin S–beta thalassemia disease, respectively. Prior to imaging, hematocrit value (HCT), systolic (SBP) and diastolic blood pressures (DBP) were measured and mean arterial pressure ($MAP = \frac{{SBP + \left( {2 \times DBP} \right)}}{3}$) was calculated for each subject.

### Image acquisition and processing

#### Vascular oxygen content

Imaging was performed by a commercially available scanning laser ophthalmoscope (Optos X200) at laser wavelengths of 532 nm and 633 nm, with a 60° field of view centered on the optic nerve head (ONH). No correction for chromatic aberrations was performed as the images at the two wavelengths appeared in good focus and registration. Retinal arteries and veins were identified by visual inspection of the appearance of blood vessels in the images acquired at the two wavelengths.

**Fig. 1:**
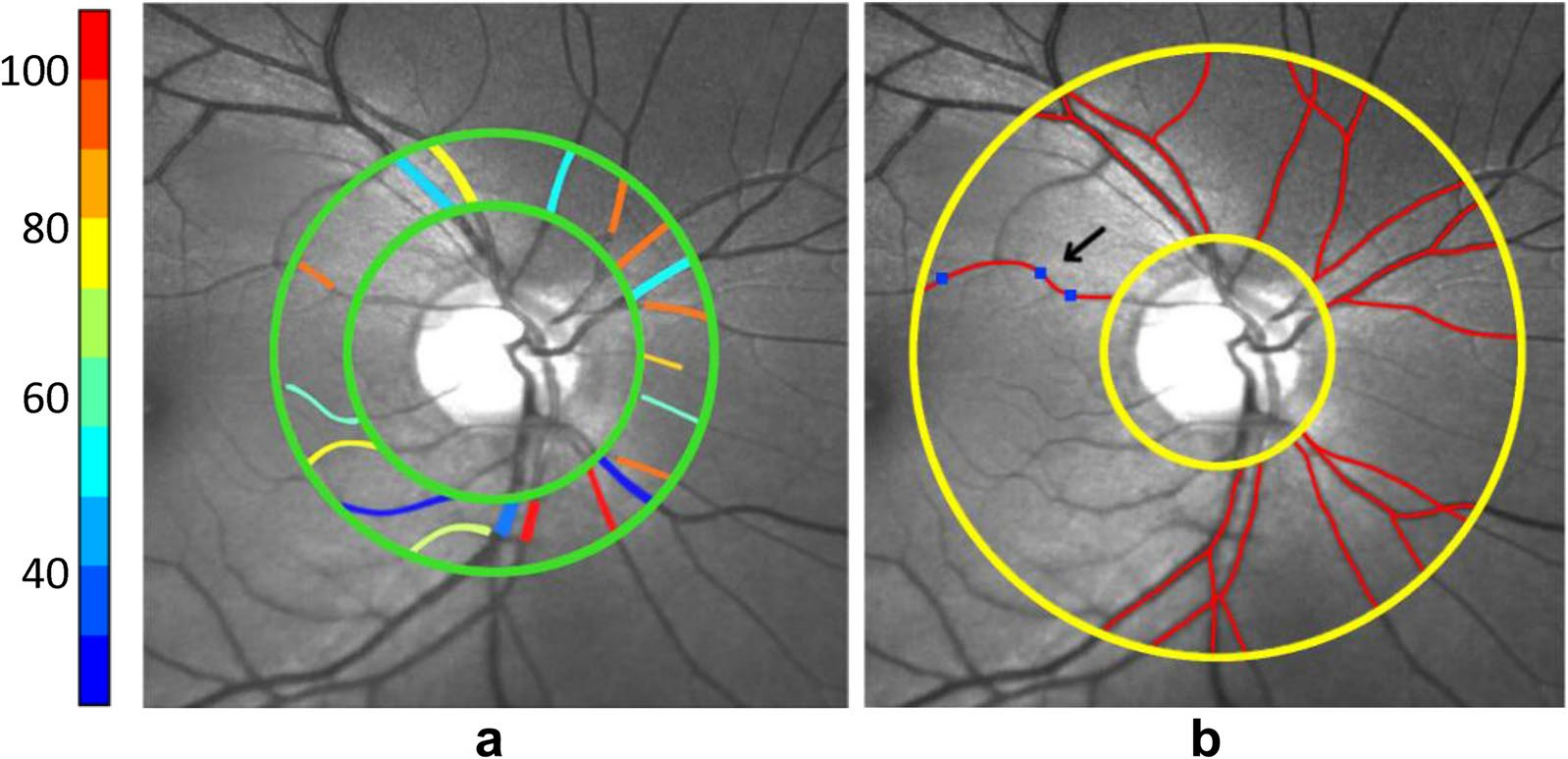
Example of a retinal image at 532 nm in a NC subject. **a** Hemoglobin oxygen saturation (SO_2_) values measured in the retinal vessel segments are displayed in pseudo color. Color bar shows SO_2_ values in percent. **b** Centerlines (red lines) of retinal vessels used for tortuosity measurements are overlaid on vessel segments. Inflection points (blue squares) for one vessel segment are indicated by the black arrow

Oxygen (O_2_) content of the retinal arteries and veins were measured using a method described by Blair et al. [14]. Briefly, a circumpapillary region centered on the ONH was defined which extended between 1 and 2 ONH radii, as shown in Fig. 1a. Retinal vasculature were detected using Frangi vesselness filter [16]. Boundary of detected vessels were determined by extracting intensity profiles perpendicular to vessel centerline every 5 pixels along the vessel and calculating the full width at half maximum of the profiles. Optical density (OD) was calculated per imaging wavelength as the average ratio of the intensity values inside to outside the vessel. Optical density ratio (ODR) was determined as OD_633_/OD_532_ and converted to SO_2_ using a linear transformation that was established for human hemoglobin oxygen saturation [17]. Figure 1a shows the circumpapillary region used for assessment of retinal vessel oxygenation together with color-coded SO_2_ measurements. Finally, using the SO_2_ measurement, O_2_ content was calculated as shown in Eq. 1.

1 $$ O_{2}  \;content = O_{2max} \times H_{g} B \cdot SO_{2} /100 $$

where $O_{2max}$ is oxygen-binding capacity of hemoglobin and $H_{g} B$ is hemoglobin concentration that was derived from HCT measurement.

#### Vessel tortuosity index

The first measure for tortuosity of retinal vessels was based on previously described vessel tortuosity index (VTI) [6]. In short, analysis was performed on the image acquired at 532 nm as it provided a higher contrast between the vessels and tissue. Measurements were obtained from a circumpapillary region centered on the ONH and extended between 1.5 and 5 ONH radii, as shown in Fig. 1b. Selection of this area was to ensure that VIT values were obtained from the same vessels in which O_2_ content was derived. Additionally, selection of a larger region allowed tortuosity measurement in extended vessel branches which are more flexible, and hence prone to tortuosity alterations. Similar to assessment of vascular O_2_ content, Frangi vesselness filtering was used for segmentation of retinal arteries and veins to provide a binary image. Vessel segmentation threshold was adjusted to exclude vessels and capillaries with diameter smaller than 25 µm. This was to match the vessels with those used for calculation of O_2_ content, and to avoid skewness of the result due to tortuosity measurements from small-caliber vessels and capillaries which are generally more tortuous than large ones [18]. Vessel endpoints were selected on the binary image and centerline extraction was performed using distance transformation. A cubic spline with a regularization parameter of 3 × 10^−5^ was used to smooth the centerline and avoid aliasing. VTI was calculated per centerline based on local and global tortuosity features as shown in Eq. (2).

2 $$ VTI = 0.1 \times \left( {SD_{\theta } \cdot N \cdot M \cdot \frac{{L_{A} }}{{L_{C} }}} \right) $$

where $SD_{\theta }$ is standard deviation of angle differences between lines tangent to each centerline pixel and the x-axis. N is number of critical points where the first derivative of the centerline vanishes. M is average ratio of centerline length to its chord length between pairs of inflection points including centerline endpoints. Finally, L_A_ and L_C_ are the length of centerline and its chord, respectively. VTI is invariant to rigid transformation and provides good correspondence with visual perception of tortuosity by human observers [6]. VTI is a unit-less quantity and has a minimum value of 0, while it has no theoretical maximum, as it increases with number of critical points and ratio of vessel length to its chord length.

#### Vessel inflection index

The second measure of tortuosity was based on vessel inflection index (VII) which is the number of inflection points along the centerline of a vessel segment. This was determined automatically for each of the extracted centerlines based on number of sign changes in the curvature of the centerline. Mathematically, these are pixels where the second derivative of the centerline vanishes. VII represents local variation along vessel segments which can differentiate between smoothly curved vessels and those with abrupt directional changes [19], and was found to be accurate for ranking tortuosity of vessels with similar length [20]. Minimum VII value is zero while there is no theoretical maximum value. Figure 1b shows the circumpapillary region used for VTI and VII analysis with vessel centerlines overlaid by red and location of inflection points for a vessel shown by blue squares.

### Statistical analysis

Demographics were compared between NC and SCR subjects using unpaired-t or Chi square tests. Retinal vascular O_2_ content, VTI and VII were averaged per eye and vessel type. Four outliers were identified and removed from the analysis. Linear mixed model analysis was performed using data obtained from both eyes of all subjects with diagnosis (NC, SCR), eye (OD, OS) and vessel type (A, V) considered as fixed effects and subjects as random effect. Shannon entropy of VTI and VII were determined to provide a level of uncertainty of each measure (from 0 for events with probability of 1 to 6.8 for all measurements with equal probability). Mutual information (MI) between the two variable was calculated to represent amount of shared information $$\left( {MI\left( {x,y} \right) = \sum\nolimits_{ij} {p\left( {{x_i},{y_j}} \right) \times \log \left( {{{p\left( {{x_i},{y_j}} \right)} \mathord{\left/
{\vphantom {{p\left( {{x_i},{y_j}} \right)} {p\left( {{x_i}} \right)p\left( {{y_j}} \right)}}} \right.
\kern-\nulldelimiterspace} {p\left( {{x_i}} \right)p\left( {{y_j}} \right)}}} \right)} } \right).$$ Mutual information is 0 for two uncorrelated measures and its maximum value is equal to entropies of two identical systems. Relationships of vascular O_2_ content with VTI and VII were determined accounting for vessel type and fellow eyes. The models were adjusted for the effects of age and MAP. Statistical tests were two-sided and significant was accepted at p ≤ 0.05.

## Results

Table 1 shows demographics of the NC and SCR subjects. Age, sex and race were similar (p ≥ 0.1), while MAP was statistically different (p = 0.01) between NC and SCR subjects. Mean and standard deviation (SD) of O_2_ content of retinal arteries and veins are shown in Tables 2 and 3, respectively. O_2_ content was lower in arteries and veins of SCR compared to NC subjects (p < 0.001). O_2_ content was higher in arteries than veins (p < 0.001), but not different between eyes (p = 0.3). VTI was not different in SCR compared to NC subjects (p = 0.2). There was no statistically significant difference in VTI between arteries and veins (p = 0.06) or between eyes (p = 0.4). VII was higher in SCR compared to NC subjects (p = 0.003). VII was higher in veins than arteries (p = 0.04), but not different between eyes (p = 0.2).

**Table 1:** Subjects’ demographics

	NC (N = 12)	SCR (N = 12)	p-value
Sex (M/F)	5/7	3/9	0.6
Race (AA/W/A/H)	7/1/1/3	12/0/0/0	0.1
Age (years)	46 ± 4	41 ± 15	0.2
MAP (mmHg)	93 ± 11	82 ± 9	0.01

AA, W, A and H stand for African American, White, Asian and Hispanic, respectively

MAP is mean systemic arterial pressure

p-values were determined by *t* test or Chi square

**Table 2:** Mean and SD of retinal arterial oxygen content (O_2A_), vessel tortuosity index (VTI) and vessel inflection index (VII) of the right (OD) and left (OS) eyes of NC and SCR subjects

Arteries	NC		SCR	
Eye	OD (N = 12)	OS (N = 12)	OD (N = 12)	OS (N = 12)
O_2A_ (mLO_2_/dL)	18 ± 3	18 ± 5	11 ± 4	12 ± 3
VTI	0.18 ± 0.06	0.15 ± 0.05	0.23 ± 0.11	0.17 ± 0.06
VII	8 ± 1	8 ± 2	10 ± 1	10 ± 2

**Table 3:** Mean and SD of retinal venous oxygen content (O_2V_), vessel tortuosity index (VTI) and vessel inflection index (VII) of the right (OD) and left (OS) eyes of NC and SCR subjects

Veins	NC		SCR	
Eye	OD (N = 12)	OS (N = 12)	OD (N = 12)	OS (N = 12)
O_2V_ (mLO_2_/dL)	13 ± 3	13 ± 3	7 ± 2	8 ± 2
VTI	0.14 ± 0.03	0.14 ± 0.03	0.16 ± 0.03	0.20 ± 0.04
VII	8 ± 1	8 ± 1	10 ± 3	9 ± 2

**Fig. 2:**
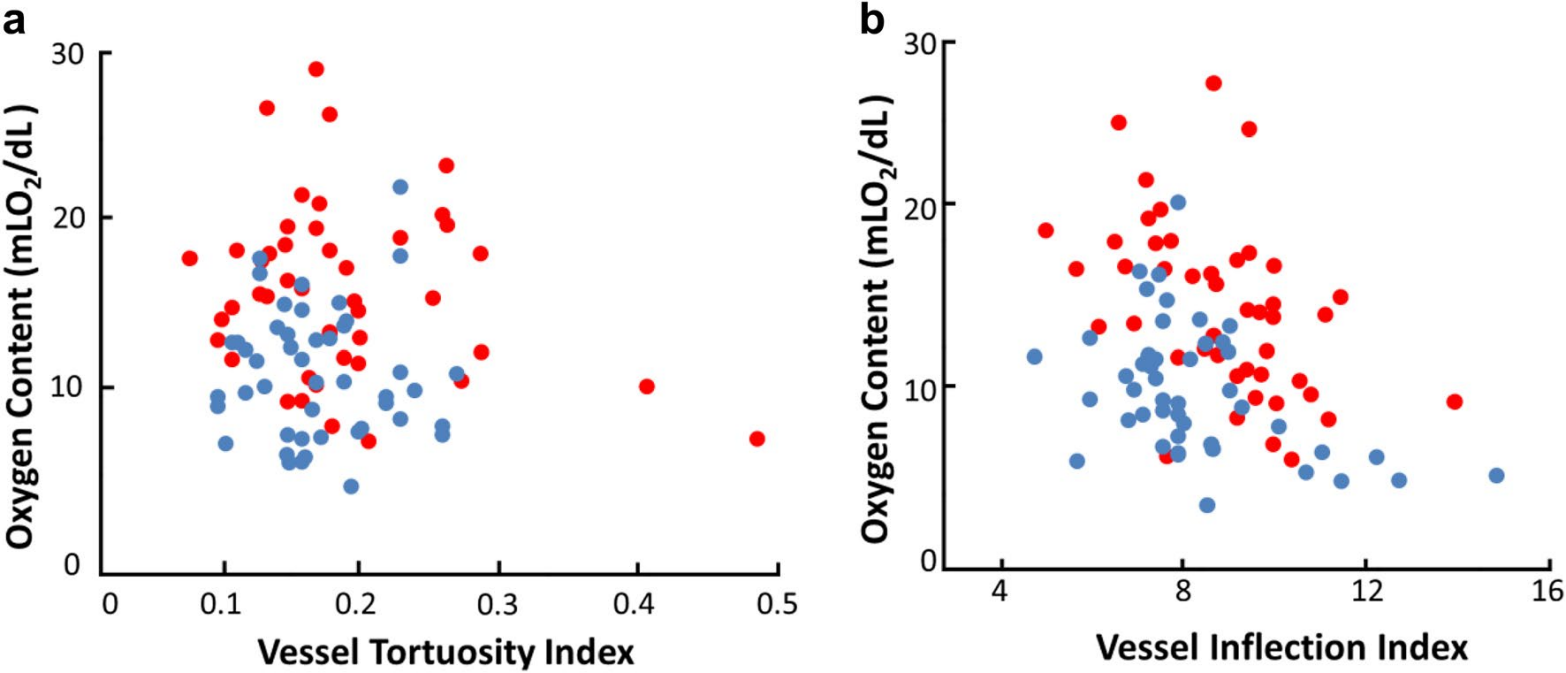
**a** Relationships of vascular O_2_ content with vessel tortuosity index (VTI) based on compiled data in NC and SCR subjects. There was no statistically significant linear relationship between O_2_ content and VIT considering both arteries and veins after adjusting for age and MAP. **b** Relationship of vascular O_2_ content with number of inflection points (VII) based on compiled data in NC and SCR subjects. There was a statistically significant negative linear relationship between O_2_ content and VII, considering both arteries and veins after adjusting for age and MAP. Data points represent measurements in retinal arteries (red dots) and veins (blue dots)

Entropy of VTI and VII were 4 and 4.3, respectively. The mutual information between the two measures were 1.3 which indicates that they are not mutually exclusive and independent. This is expected since VTI and VII were calculated based on tortuosity features of the same vessel segments. Figure 2a shows the relationship between O_2_ content and VTI in retinal arteries and veins based on compiled data in all the subjects. There was no statistically significant linear relationship between O_2_ content and VTI (p = 0.3, β = −5.7). Figure 2b shows the relationship between O_2_ content and VII in retinal arteries and veins based on compiled data in all the subjects. There was a statistically significant negative linear relationship between O_2-_ content and VII (p = 0.02, β = −0.5).

## Discussion and conclusion

Although previous studies have reported alterations in retinal vessel tortuosity and vascular oxygen content due to SCR [4, 6–8], a relationship between these metrics has not been established. Due to a lack of formal definition of vessel tortuosity and disease-specific variations in vessel features that contribute to overall tortuosity features [21], two different measures of vessel tortuosity were used in the analysis to increase reliability of the results. In the current study, we confirmed the hypothesis that oxygenation of retinal vasculature is inversely related with increased retinal vessel tortuosity. We found no statistically significant association between vascular O_2_ content and VTI. However, we found that reduced vascular O_2_ content was significantly associated with increased VII or number of inflection points along retinal vessels, suggesting retinal vessels become more undulated in response to reduced oxygenation.

Measurements of retinal vascular O_2_ content of NC subjects in the current study was consistent with previously reported values [4, 22]. Additionally, reduced O_2_ content of retinal arteries and veins in SCR subjects was in agreement by our previous study which used a different oximetry instrument [4]. To our knowledge, there is no quantitative report on tortuosity of the main branches of central retinal artery and vein in ONH region of SCR subjects. The finding of increased VII in larger retinal vessels near the ONH is consistent with reported increased vessel tortuosity in smaller retinal vessels in the macular region [6, 7, 23, 24], suggesting similarity between morphological changes according to vessel caliber and retinal regions in SCR. The significant difference in VII between arteries and veins are in accordance with previous studies which suggested a difference in tortuosity due to type of retinal vessel [25]. This is because veins are generally more flexible than arteries [26, 27]. Finally, the finding of no statistical differences between the fellow eyes is consistent with previous reports [28, 29]. To avoid data redundancy and overestimation of difference between the subjects, the correlation effect of using data from both eyes was controlled as a covariate in the current analysis.

The inverse linear relationship between retinal vascular O_2_ content and VII implies that the number of inflection points is increased with reduced oxygenation of retinal vasculature. This finding is supported with previous report of increase in retinal arterial tortuosity as an early outcome of oxygen induced retinopathy in mice [13]. An undulated vessel is longer than a straight one [9], and hence covers a larger region of the retina, which may allow higher oxygen diffusion to the tissue. In fact, O_2_ is released to the tissue due to pressure gradient caused by difference in partial pressure between O_2_ and carbon monoxide (CO_2_) [30]. Decrease in O_2_ content reduces the gradient of the pressure, and hence limits the volume and extent of oxygen delivery to the tissue. To compensate, vessels may tend to become undulated to cover expanded region to maintain sufficient tissue oxygenation. This finding is in accord with a previous study which suggested retinal vessels become undulated, at least in part, to compensate for neuro-retinal hypoxia due to diabetes [31]. It was also suggested that undulation of a vessel along its course can predict incident of diabetic retinopathy in Type I diabetes [31].

VTI was not different between NC and SCR subjects, neither between arteries and veins. The lack of statistical difference in VTI may indicate that this measure of tortuosity is not sensitive enough to demonstrate tortuosity alterations in the selected region. However, we showed previously that VTI can detect statistical difference in tortuosity of retina vessels of SCR subjects in the macular region imaged by OCTA [6]. Future studies are needed to determine sensitivity of VTI for detection of tortuosity alterations in larger retinal vessels. Our results did not show a linear relationship between VTI and O_2_ content in major retinal vessels. Future studies in a larger cohort and broader retinal regions are needed to detect changes in VTI of retinal vessels and establish a potential relationship with oxygenation.

The current study had limitations. First, oxygen hemoglobin dissociation curve may be different between NC and SCR subjects which may have affected the calculation of O_2_ content. However, previous studies have shown that the difference in SO_2_ values larger than 92% is small and the curve is skewed toward the right for smaller values [32]. Also, there might be a difference in hemoglobin absorption spectra between NC and SCR subjects as it was shown using blood samples [33, 34]. Second, there were variations in disease stage and genotype of SCR subjects which necessitates future studies in a more homogeneous cohort. However, the majority of subjects had stage II retinopathy (92% of SCR subjects) and had SS hemoglobin (67% of SCR subjects) disease. Finally, in some subjects, O_2_ content and tortuosity measurements were obtained from different vessels. Nonetheless, the measurements were averaged per eye to derive a representative value. Future studies in a larger cohort are needed to confirm the findings and better characterize the nature of the relationship between retinal vascular oxygenation and tortuosity metrices and evaluate the effect of vessel size and retinal region on these relationships. This is important because SCD subjects demonstrate varying retinal pathology with heterogeneous phenotypic expression even in subjects with the same genotype [35].

Overall, the current study showed an inverse linear relationship between retinal vascular oxygen content and a vessel tortuosity metric. The findings contribute to our understanding of retinal pathophysiology and may provide vascular morphological biomarkers for assessment of retinal hypoxia due to SCR and other retinopathies.

## Data Availability

The data that was used in the current study are not publicly available due to privacy and security of patients.
